# The Vaccination of 35,000 Dogs in 20 Working Days Using Combined Static Point and Door-to-Door Methods in Blantyre, Malawi

**DOI:** 10.1371/journal.pntd.0004824

**Published:** 2016-07-14

**Authors:** Andrew D Gibson, Ian G Handel, Kate Shervell, Tarryn Roux, Dagmar Mayer, Stanford Muyila, Golden B Maruwo, Edwin M. S Nkhulungo, Rachel A Foster, Patrick Chikungwa, Bernard Chimera, Barend M.deC Bronsvoort, Richard J Mellanby, Luke Gamble

**Affiliations:** 1 Mission Rabies, Cranborne, Dorset, United Kingdom; 2 The Roslin Institute and The Royal (Dick) School of Veterinary Studies, Division of Genetics and Genomics, The University of Edinburgh, Hospital for Small Animals, Easter Bush Veterinary Centre, Roslin, Midlothian, United Kingdom; 3 Blantyre Society for the Protection and Care of Animals, Blantyre, Malawi; 4 Department of Animal Health and Livestock Development, Blantyre Agriculture Office, Blantyre, Malawi; 5 Blantyre Regional Veterinary Laboratory, Blantyre, Malawi; 6 Department of Animal Health and Livestock Development, Blantyre Agriculture Development Division, Blantyre, Malawi; 7 Sheffield Teaching Hospitals NHS Foundation Trust, Department of Infectious Diseases and Tropical Medicine, Royal Hallamshire Hospital, Sheffield, United Kingdom; 8 Department of Animal Health and Livestock Development, Lilongwe, Malawi; 9 The Roslin Institute and The Royal (Dick) School of Veterinary Studies, Division of Veterinary Clinical Studies, The University of Edinburgh, Hospital for Small Animals, Easter Bush Veterinary Centre, Roslin, Midlothian, United Kingdom; Wistar Institute, UNITED STATES

## Abstract

An estimated 60,000 people die of rabies annually. The vast majority of cases of human rabies develop following a bite from an infected dog. Rabies can be controlled in both human and canine populations through widespread vaccination of dogs. Rabies is particularly problematic in Malawi, costing the country an estimated 13 million USD and 484 human deaths annually, with an increasing paediatric incidence in Blantyre City. Consequently, the aim of this study was to vaccinate a minimum of 75% of all the dogs within Blantyre city during a one month period. Blantyre’s 25 administrative wards were divided into 204 working zones. For initial planning, a mean human:dog ratio from the literature enabled estimation of dog population size and dog surveys were then performed in 29 working zones in order to assess dog distribution by land type. Vaccination was conducted at static point stations at weekends, at a total of 44 sites, with each operating for an average of 1.3 days. On Monday to Wednesday, door-to-door vaccination sessions were undertaken in the areas surrounding the preceding static point stations. 23,442 dogs were vaccinated at static point stations and 11,774 dogs were vaccinated during door-to-door vaccinations. At the end of the 20 day vaccination programme, an assessment of vaccination coverage through door-to-door surveys found that of 10,919 dogs observed, 8,661 were vaccinated resulting in a vaccination coverage of 79.3% (95%CI 78.6–80.1%). The estimated human:dog ratio for Blantyre city was 18.1:1. Mobile technology facilitated the collection of data as well as efficient direction and coordination of vaccination teams in near real time. This study demonstrates the feasibility of vaccinating large numbers of dogs at a high vaccination coverage, over a short time period in a large African city.

## Author Summary

Rabies is a devastating disease that is estimated to result in the death of approximately 60,000 people every year. Most humans contract rabies following a bite from an infected dog and it has been demonstrated that provided a large enough proportion of the dog population is vaccinated; rabies incidence can be markedly reduced in both humans and dogs. However, few studies have described the vaccination of large numbers of dogs in a short period. An increasing incidence of childhood rabies has been reported in Blantyre, Malawi’s second largest city, over the past decade. This study reports the development and implementation of a mass canine rabies vaccination campaign which used a combined approach of static point and door-to-door vaccination strategies. 23,442 dogs were vaccinated at static point stations and 11,774 dogs were vaccinated during door-to-door vaccination during 20 working days of vaccination. Post vaccination surveys revealed vaccine coverage of 79.3% (95% CI 78.6–80.1). This study, through the unique approach of using novel mobile technology and two vaccination strategies, demonstrates the feasibility of vaccinating over 35,000 dogs within a one month period in an African city where rabies has a high prevalence in the human population.

## Introduction

Rabies is a devastating disease which is estimated to kill approximately 60,000 people a year [1]. Globally, rabies is estimated to cause 3.7 million disability-adjusted life years and results in 8.6 billion US dollars of economic losses annually [1]. Successful treatment of patients with clinical signs related to rabies infection has only rarely been reported and case fatality approaches 100% [2]. Dogs are the principal reservoir for human rabies and are responsible for almost all human cases [2]. Consequently, controlling rabies in dogs is considered critical in the quest to eliminate rabies in both canine and human populations [3,4]. The mass vaccination of dogs has been successful in reducing the incidence of rabies in humans and dogs in many parts of the world, notably Central and South America [5]. However, attempts to eliminate rabies from Africa have been much less successful with the highest per-person death rate occurring in the poorest countries in sub-Saharan Africa [1].

One of the greatest challenges to the implementation of canine rabies vaccination programmes is ensuring that a high proportion of all owned and free-roaming dogs are vaccinated. Vaccination coverage of at least 70% has been demonstrated to sufficiently reduce transmission to control outbreaks of disease in dog populations [4,6–8] and the importance of achieving even coverage across the target area has been recently highlighted [9]. Whilst there have been occasional examples of mass vaccination in rabies endemic areas in Africa which have reduced the incidence of the disease in humans and dogs, notably in KwaZulu-Natal, South Africa [10] and Serengeti, Tanzania [11], many vaccination programmes have either failed to vaccinate large numbers of dogs or have failed to achieve vaccination coverage of over 70% [4,7].

In an effort to increase the number and proportion of dogs vaccinated in Africa, several vaccination approaches have been reported including house-to-house visits, fixed vaccination posts in well recognised sites within a community and temporary vaccination posts set up by mobile teams. In isolation, static point vaccinations arrangements, which are widely used in canine rabies vaccination programmes in Africa, are often unable to reach a high enough proportion of dogs in all community settings [11–14]. In addition, door-to-door programmes where a vaccination team visits every house within the catchment area, can be logistically challenging to run and are invariably more expensive and labour intensive [12]. Combined door-to-door and static point vaccination approaches have been demonstrated to be effective where static point methods alone have failed to reach sufficient coverage [12]. However, reports of studies using multiple approaches to ensure high vaccination coverage of a large number of dogs in Africa are particularly scarce.

Malawi faces many challenges to development, with a high human population density, one of the world’s highest incidences of HIV [15], and a severely under resourced health services. In 2012 Depani et al reported a threefold increase in the number of paediatric rabies cases presenting to The Queen Elizabeth Central Hospital in Blantyre since the period 2002–2005 [16], with many rabies cases going undiagnosed or misdiagnosed [17]. Post exposure prophylaxis is often in short supply and 3 in every 100,000 people in Malawi are estimated to die of rabies annually, costing the country an estimated 13 million USD per year in direct cost of post-exposure prophylaxis (PEP), and indirect costs of seeking PEP, lost income, productivity losses from premature deaths, dog vaccination and population management, livestock losses and surveillance [1].

Innovation in the use of mobile technology to enhance data collection and near real- time monitoring of disease interventions and population surveillance has been growing in recent years [18–20]. Mission Rabies have been using a tailor made smartphone application to direct and monitor vaccination teams in India [21], however the use of mobile technology to improve methods of mass dog vaccination in Africa has been limited to date.

The aim of this study was to investigate the feasibility of rapidly vaccinating a high proportion of dogs across an African city with a large dog population and a high prevalence of human rabies and marks the start of a three year programme to eliminate rabies in Blantyre District.

## Methods

### Study site

**Fig 1:**
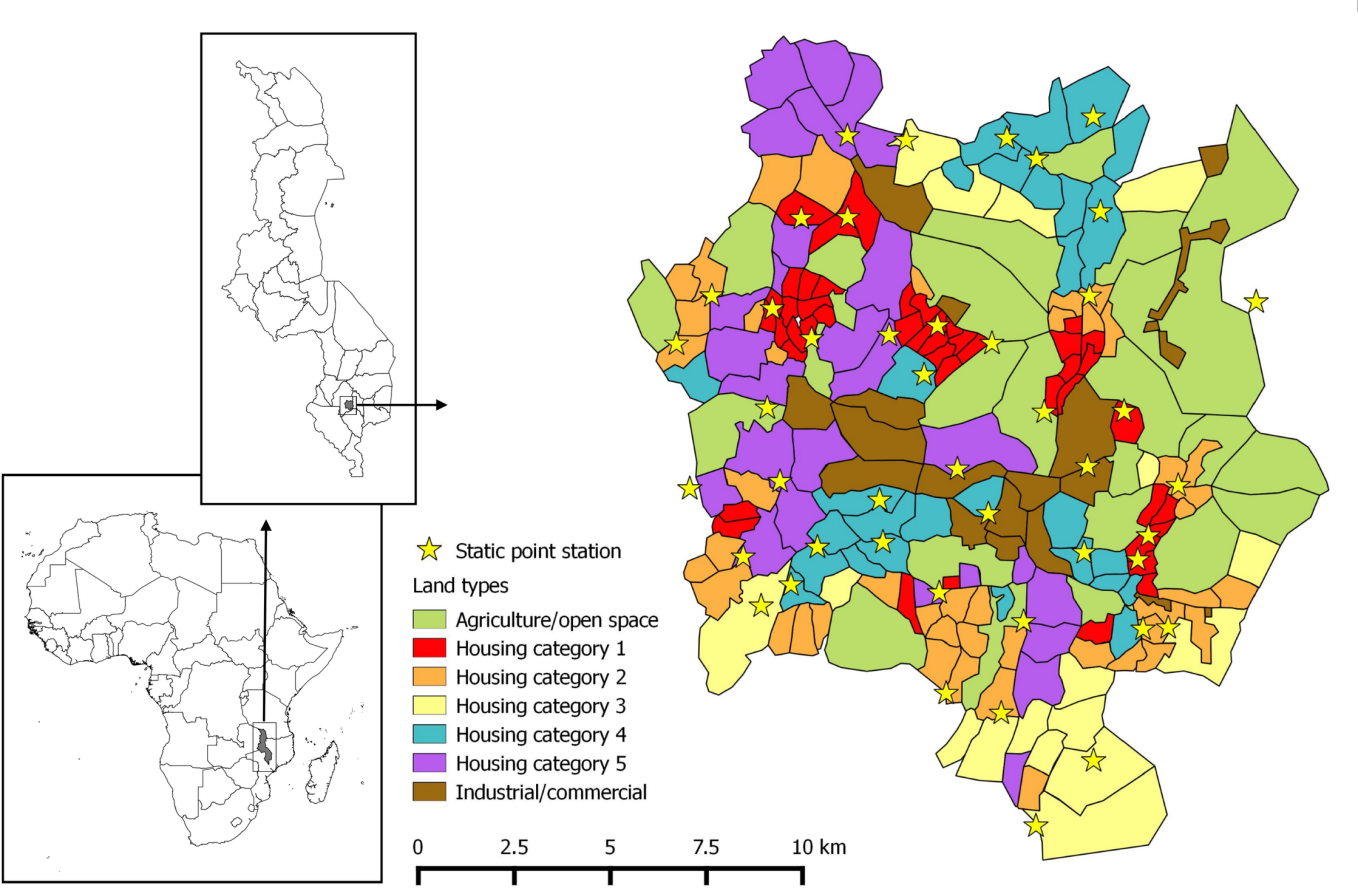
Map of the study site. Inset map of Africa showing location of Malawi and inset of Malawi showing location of the city of Balantyre. Main map showing Blantyre city and distribution of different housing categories and static point vaccination locations.

Blantyre City is the second largest city in Malawi with a human population of 825,000 people (based on 3.2% annual growth rate since the 2008 census [22]). It lies within the larger administrative region of Blantyre District which has an estimated canine population of 73 400 dogs [23]. The city is divided into 25 administrative wards and land use across the city is diverse, with industrial, agricultural, residential, mountainous and open land all being present to varying degrees within wards. The city was divided into 204 working zones to facilitate field work using open source Google My Maps. Zones were sized subjectively, based on the estimated area that a vaccination or survey team could cover on foot within one day and were imported into QGIS Desktop 2.6.1 (QGIS development team, Open Source Geospatial Foundation Project) for calculation of area. The working zones were stratified into one of seven land types based on their appearance in Google Satellite images; housing category 1 (small houses–high density); housing category 2 (small houses–low density); housing category 3 (small houses–low density); housing category 4 (medium houses–ordered); housing category 5 (large houses–medium/low density); industrial/commercial; agriculture/open space (Fig 1).

### Mobile technology

**Fig 2:**
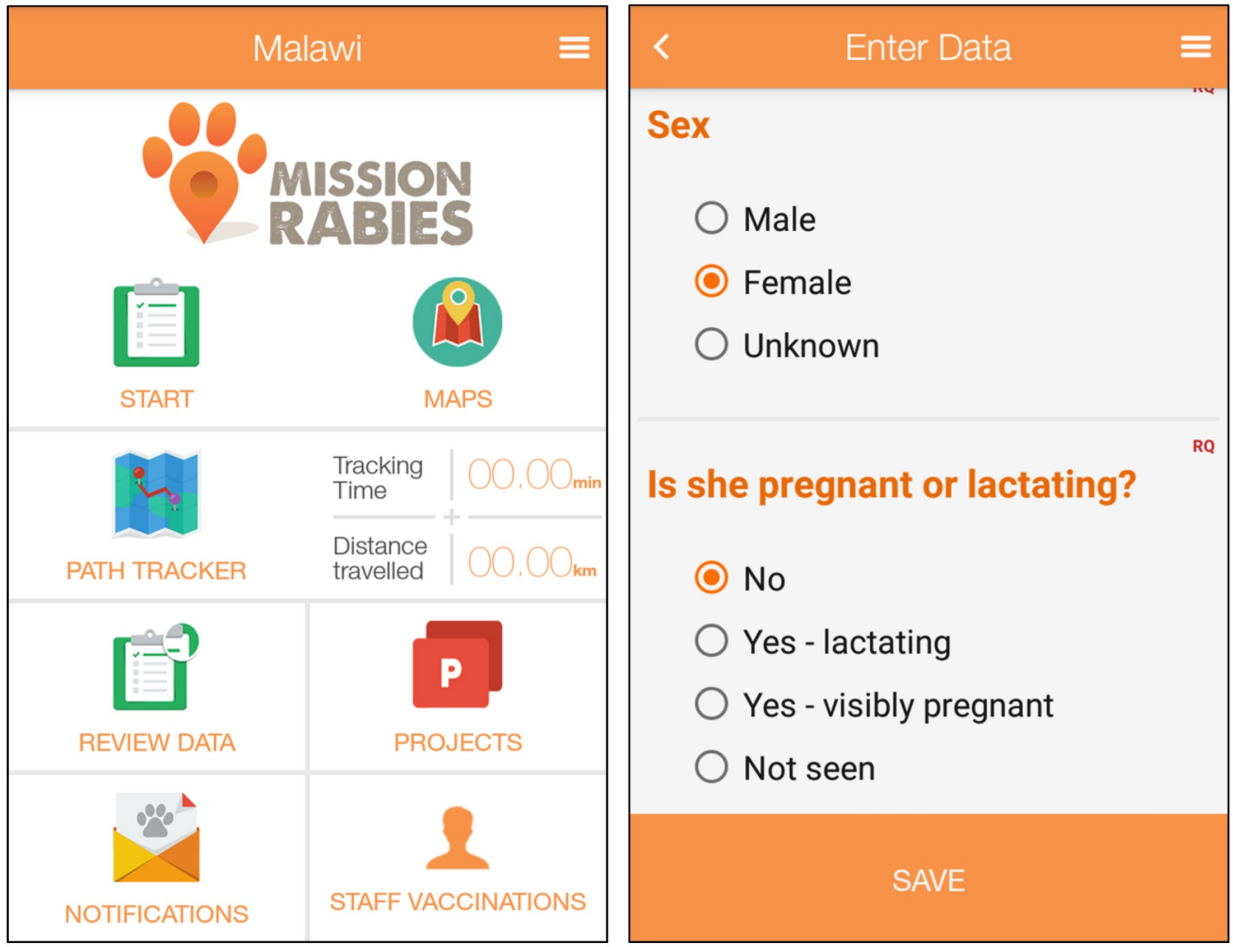
Screenshots from the Mission Rabies App. Left: home screen from which the user can navigate to various App functions. Right: data entry screen, scroll function allows for rapid data entry.

Project management was facilitated through use of a smartphone application (Mission Rabies App) and web-based backend platform, tailored towards management of mass dog vaccination work. Smartphone handsets (Samsung Galaxy J1) with the Mission Rabies App installed were used by teams in the field to enter vaccination and survey data, to navigate within demarcated boundaries during roaming work and to share key information such as contact phone numbers and real-time team locations (Fig 2). The dates of surveys, vaccinations and suspected rabies cases were recorded on the phones using questionnaire forms, pre-designed by an administrator on the backend platform and remotely loaded to the handsets using 3G.

**Fig 3:**
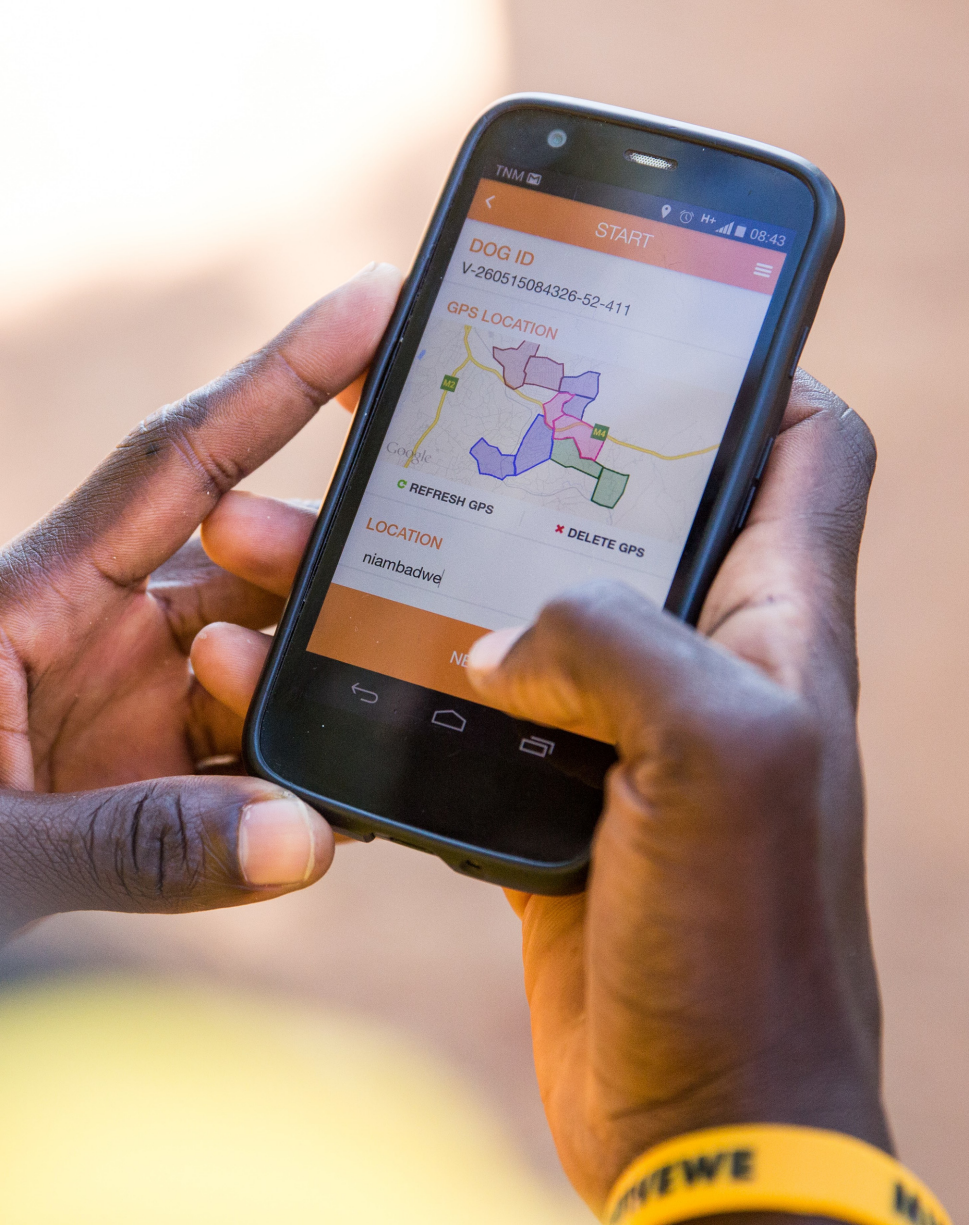
Working boundaries with team colours displayed on handset in the field.

Data were entered offline and stored locally on the handset where it could be reviewed on a map. At the end of a vaccination session or survey, and once a secure internet connection was available, the data was uploaded to a cloud-based server where it could be accessed online from the web backend. Crucially, geographic working boundaries were present on the App itself which greatly facilitated navigation in areas which had unmarked or unofficial roads and paths, had no discernible street names or were not accurately represented on the available paper-based maps of the city. A project manager assigned the working zone for each door-to-door vaccination team and demarcated the boundaries for each zone in an individual shaded colour on the App backend platform the day before. These boundaries were then automatically synchronized to the App on each teams’ handset via Wi-Fi or 3G connection, with each team assigned a different colour (Fig 3). The teams then navigated through their zone using the map on the phone whilst entering data in the field. To save battery life, or in areas of poor internet connectivity, the App allowed the teams to work offline and still navigate using the maps function. A live path-tracking function on the App also meant that the teams could regularly review the areas they had already covered, so as to ensure that the entire zone could be reached as efficiently as possible, particularly where the teams were not familiar with the area that they were working in. In case of any technical issue with the smartphone data collection, the same information was recorded using paper records with manual entry of the vaccination location instead of GPS and subsequently added to the dataset manually.

### Population assessment

For the purpose of vaccine procurement, budgeting and initial planning, the dog population of Blantyre was estimated using a mean African urban human:dog ratio of 21.2:1 from the literature [24]. In the month prior to the start of the vaccination campaign, a dog sight survey was conducted from 31^st^ March to 20^th^ April 2015 in order to further assess dog population size and distribution across the city. Twenty nine of the 204 working zones were randomly selected on a proportionate basis from within each of the 7 land types using a random number generator. Surveys were conducted between 6am and 9am by individuals walking through a region on foot, attempting to survey every public area including roads, alleys and side streets. Dogs sighted were recorded on the Mission Rabies App with the dataset for each dog including GPS, confinement status at the time of sighting (free roaming/ tied on property/ in enclosed yard/ inside house), sex, age and the lactation status of females (adult male/ adult non-lactating female/ adult lactating female/ adult unknown sex/ puppy). Throughout the study, free roaming dogs were defined as any roaming dog that was not confined to private property, restrained by a leash or chain or under direct human control at the time of sighting. The precise GPS path walked by surveyors was also recorded and synchronized to the cloud-based server through the App. The area surveyed was estimated using the survey path-tracker, allowing the mean number of dogs sighted per square kilometre to be calculated and used to extrapolate for un-surveyed areas. To estimate roaming dog density for each land type, the total number of free roaming dogs sighted in each survey for a land type was assumed to be Poisson distributed with a mean *d_i_* × *A_i_* where *d_i_* is the density of dogs in area *i* and *A_i_* is the total land area of area *i*. Density was assumed to be constant for each land type. A Bayesian framework was used to estimate the densities for each land type using the JAGS [25] software tool in the R statistical software environment [26] using the package rjags [27] providing posterior means and 95% credible intervals for density and total free roaming dog numbers (using simple multiplication by total land areas) with a vague (Gamma (0.001, 0.001)) prior distribution for dog density.

### Vaccination

Vaccination was carried out across the city in a systematic fashion using a combination of static point and door-to-door vaccination strategies. There were eight vaccination teams working simultaneously, with each team consisting of two vaccinators, two data collectors and two animal handlers/certificate writers. Static point vaccination stations were conducted at weekends, followed by mobile vaccination teams travelling door-to-door on Monday, Tuesday and Wednesday in the working zones surrounding those static point stations. A staff induction session, including rabies related educational talks and staff participation was conducted on 26^th^ April, followed by a door-to-door vaccination training day on 29^th^ April 2015. The vaccination campaign ran from 29^th^ April to 27^th^ May 2015. During this time, vaccinations were performed over 20 full working days (including two half days).

Vaccinations were provided free of charge and each dog was administered with a dose (1ml) of rabies vaccine (Nobivac Rabies–MSD Animal Health), either subcutaneously or intramuscularly. Dogs of all ages were vaccinated and marked on the forehead with a brightly coloured livestock marker (Red or Green “Top Marker”. Kerbl, Albert Kerbl GmbH, Felizenzeil 9, 84428 Buchbach, Germany. www.kerbl.de) to allow their vaccinated status to be readily identifiable for several days after vaccination. Each owner was issued with a vaccination certificate, wrist band and rabies awareness educational leaflet in the local language.

### Static point vaccination

The static point vaccination stations were advertised during the preceding weeks on posters and radio, as well as in the preceding days using loud speaker announcement in the streets. A meeting of all ward councillors was convened to inform them of the objectives of the programme and of the dates of the vaccination stations so that they could mobilise their respective ward communities. For stations with a higher anticipated turnout, two vaccination teams were posted and in some cases a single team returned to the same station for a second day. Data about each dog was recorded on the Mission Rabies App, including GPS location at the point of vaccination, sex, signs of lactation or pregnancy (if female), neuter status, age (more than or less than 3 months), skin condition on a four point score (1 no skin disease/ 2 mild (<20% of the body affected)/ 3 moderate (20–80% of the body affected)/ 4 severe (>80% of the body affected)), body condition score (1 emaciated/ 2 underweight/ 3 ideal condition/ 4 overweight), presence of other health conditions (TVT or genital mass/ wounds/ lameness/ ear lesions/ other (free text entry)). Finally there was a free text area for additional comments about individual animals. Dogs vaccinated at static point clinics were all assumed to be ‘owned’ due to the fact that someone had taken responsibility for presenting the dog for vaccination.

### Door-to-door vaccination

The same teams working together at the weekend in static point stations formed the door-to-door vaccination teams the following week. Each team often divided into two sub-teams, each consisting of one vaccinator, one data collector and one animal handler to coordinate vaccination across the designated area. The teams carried a field kit consisting of vaccines within a cool-bag, a butterfly net to assist in the restraint of aggressive dogs and a bag carrying general supplies, including syringes, needles, vaccination certificates, wristbands, stock-marker and information leaflets. All dogs encountered during door-to-door vaccination were recorded. This included previously unvaccinated dogs which the team vaccinated during the door-to-door vaccination approach, dogs marked as vaccinated that had previously presented to a static point station (or had a correlating certificate) and unmarked dogs seen, but could not be vaccinated for any reason (recorded as unable to catch or restrain/ owner refused/ owner not present to give consent). Through the collection of data on all dogs encountered, the door-to-door work also served as a survey of regions for vaccination coverage achieved from static point vaccination alone.

For dogs vaccinated during the door-to-door vaccination approach, in addition to the data collected as per the static point method, ownership status and current level of confinement (free roaming/ confined on property/ confined inside house) were also recorded. A dog was recorded as “Identifiably owned” if any signs of ownership were observed, including the dog wearing a collar, an owner being present or a resident saying that the dog was owned by someone in the community. Both identifiably owned and unowned dogs were vaccinated during door-to-door work, with the use of butterfly nets to assist in the capture of unapproachable free roaming dogs. A chi squared test with Yates correction was used to test for differences in distribution in body condition scores and reproductive status for dogs encountered at door-to-door vaccination.

### Assessment of vaccination coverage

After the 20 day vaccination programme was completed, door-to-door household questionnaire surveys were conducted through randomly selected residential working zones (Housing category 1–5) in the two weeks following the end of the campaign. By this time temporary marks applied at vaccination would have faded and so dog sight surveys could not be used. Land types open space/agriculture and commercial/industrial areas were not included as for the most part they were uninhabited or not accessible for survey. The surveyors were instructed to walk zig-zag transects through each working area, enquiring at every house encountered and recording whether any dogs were owned or not. For dog-owning households, the number of adults and puppies was recorded, including how many of each had been vaccinated and, of these, whether they were vaccinated by the Mission Rabies project. Owners were required to present a valid vaccination certificate to confirm vaccination and vaccination coverage was calculated as the proportion of vaccinated dogs.

Survey data were labelled by land type according to GPS and working zone using QGIS. Subsequent calculation of vaccination coverage was done by land type within each survey. Binomial exact confidence intervals were calculated for each post-vaccination survey.

After the campaign a city wide owned dog population estimate was calculated by grouping post vaccination survey data and using the Lincoln-Petersen’s formula [28];

$$N=\frac{\left(n_{1}+1)(n_{2}+1\right)}{\left(m_{2}+1\right)}-1$$

Where N is the total estimated population size, n_1_ is the number initially vaccinated, n_2_ is the total number of dogs recorded on post vaccinations survey and m_2_ is the number of vaccinated dogs recorded on post vaccination surveys. Approximate 95% confidence intervals were calculated using the Seber’s formula, used by Tenzin et al (2015) [29].

$$var=\left[\frac{\left(n_{1}+1)(n_{2}+1)(n_{1}-m)(n_{2}-m\right)}{{\left(m+1\right)}^{2}(m+2)}\right]$$

The 95% confidence interval for N was estimated as

$$N\pm 1.965\sqrt{var(N)}$$

The total dog population was then adjusted for the estimated proportion of unowned dogs encountered during door-to-door vaccination.

### Ethics statement

Prior to vaccination of owned dogs, verbal informed consent was obtained from the person presenting the dog for vaccination. In the cases where an owner could not be identified, dogs were vaccinated in accordance with Government Public Health protocol, as the work was part of a non-research public health campaign.

## Results

### Population assessment

Total dog population was estimated at 38,897 using the literature human:dog ratio. Small houses at high and medium density were the most common land type classifications (Table 1). Few dogs were sighted in agriculture and open space land types whereas the highest density of dogs was sighted in the high density small house classification (Table 1). One of the three designated open space/agricultural areas was completely inaccessible for survey.

**Table 1:** Stratification of working zones and population counts in representative surveys. Housing category 1 (Small houses—high density), Housing category 2 (Small houses—medium density), Housing category 3 (Small houses—low density), Housing category 4 (Medium houses–ordered), Housing category 5 (Large houses—medium/low density). S1 Dataset gives the breakdown of city zones and dogs sighted by zone in surveyed regions.

Land type	Total number of zones	Number of surveys	Mean free roaming dogs sighted per Km^2^ (95% CI)	Extrapolated free roaming population within strata (95%CI)
Agriculture/open space	21	2	5.6 (3.2–8.7)	384 (221–593)
Housing category 1	46	8	473.2 (435.9–512.7)	7,822 (7206–8475)
Housing category 2	44	6	234.9 (209.9–260.8)	6997 (6251–7768)
Housing category 3	19	2	12.9 (6.7–21.2)	523 (271–859)
Housing category 4	28	4	81.7 (69.1–85.4)	2,485 (2104–2901)
Housing category 5	27	4	11.0 (7.6–14.9)	471 (327–639)
Industrial/commercial	19	3	3.6 (1.8–6.0)	81 (41–135)
**Total**	**204**	**29**		**18,763 (17,650–19,919)**

The pre-vaccination survey provided an estimate of 18,763 free roaming dogs and revealed that ‘small houses-high density’ and ‘small houses-medium density’ land types had the highest density of dogs. This information allowed the vaccination teams to be proportionately deployed according to predicted density of dogs.

### Vaccination

During the 20 working days of the vaccination programme, 35,216 dogs were vaccinated (Table 2). There were 44 static point stations at which 23,442 dogs were vaccinated, whilst a further 11,774 dogs were vaccinated by door-to-door vaccination teams. Many open space/agricultural and some industrial/commercial areas were not accessible to vaccination teams on foot.

**Table 2:** Table of records from static point and door-to-door vaccination for all data captured during the campaign, including dogs vaccinated by static point and door-to-door activities, already vaccinated dogs encountered at door-to-door and dogs sighted but not vaccinated .

Activity	Type of record	Number of dogs
Static point records	Vaccine administered	23442
Door-to-door records	Vaccine administered	11774
	Already vaccinated at static point	9581
	Dog sighted, but not vaccinated	1268
**Total records**		**46065**
**of which vaccinations**		**35216**

During door-to-door vaccination work, 42.4% of dogs were identified as having been vaccinated at static point vaccination stations held the previous weekend within the same area. Of the remaining unvaccinated dogs sighted 90.3% could be restrained for vaccination. Reasons for not vaccinating dogs by door-to-door teams included; unable to restrain (54.5%), owner not present (30.5%), owner refused (8.2%), already vaccinated by a non-Mission Rabies source (5.3%), other (1.4%). Of all dogs recorded during door-to-door vaccination 97.1% were identifiably owned, however 56% of “unowned” dogs were marked with paint from a static point clinic, indicating that they had been presented by someone for vaccination. It was possible to catch and vaccinate 38.6% of unmarked, unowned roaming dogs sighted at door to door vaccination. A technical problem meant that 365 records were recorded on paper at static point stations during one morning of the programme. These records were added by hand to the master database. S2 Dataset gives a breakdown of vaccination data and tables summarising calculations.

### Vaccination coverage

**Fig 4:**
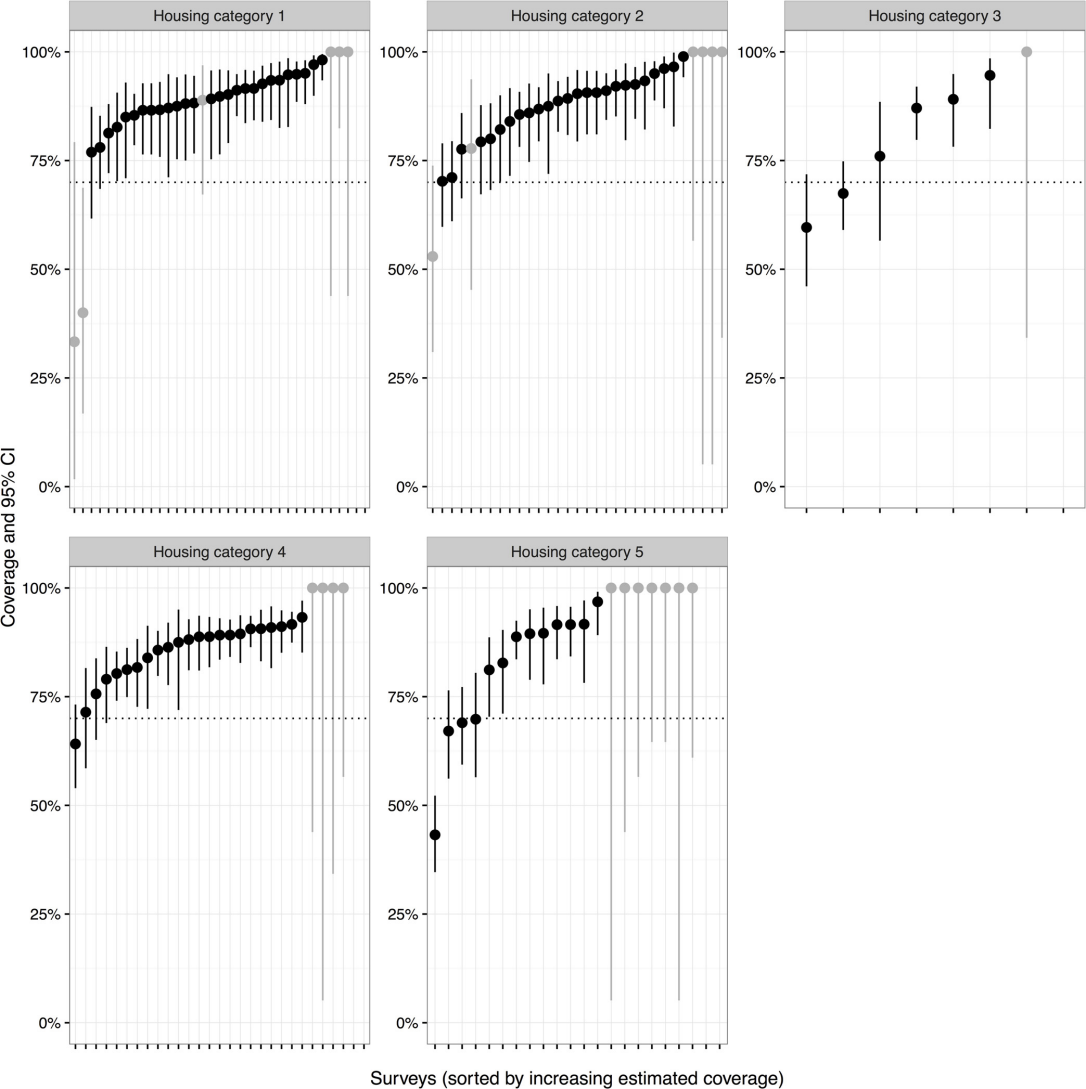
Graphs of estimated vaccination coverage for adult dogs by individual post vaccination survey and grouped according to land type (Housing categories 1–5). The 95% confidence intervals for each survey are shown. Light grey lines indicated surveys which recorded less than 20 dogs within that particular land type.

**Fig 5:**
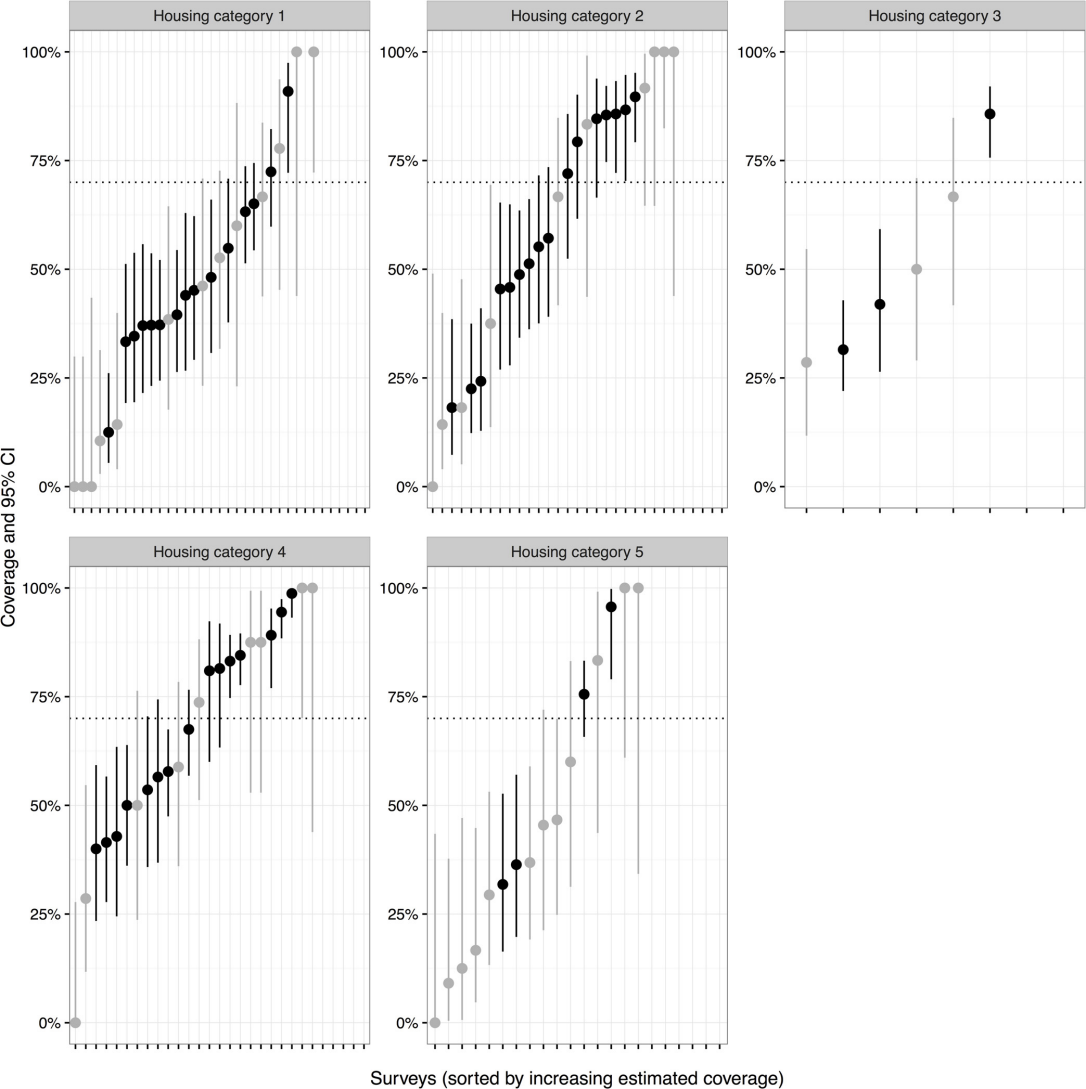
Graphs of estimated vaccination coverage for puppies by individual post vaccination survey and grouped according to land type (Housing categories 1–5). The 95% confidence intervals for each survey are shown. Light grey lines indicate surveys which recorded less than 20 dogs within that particular land type.

**Fig 6:**
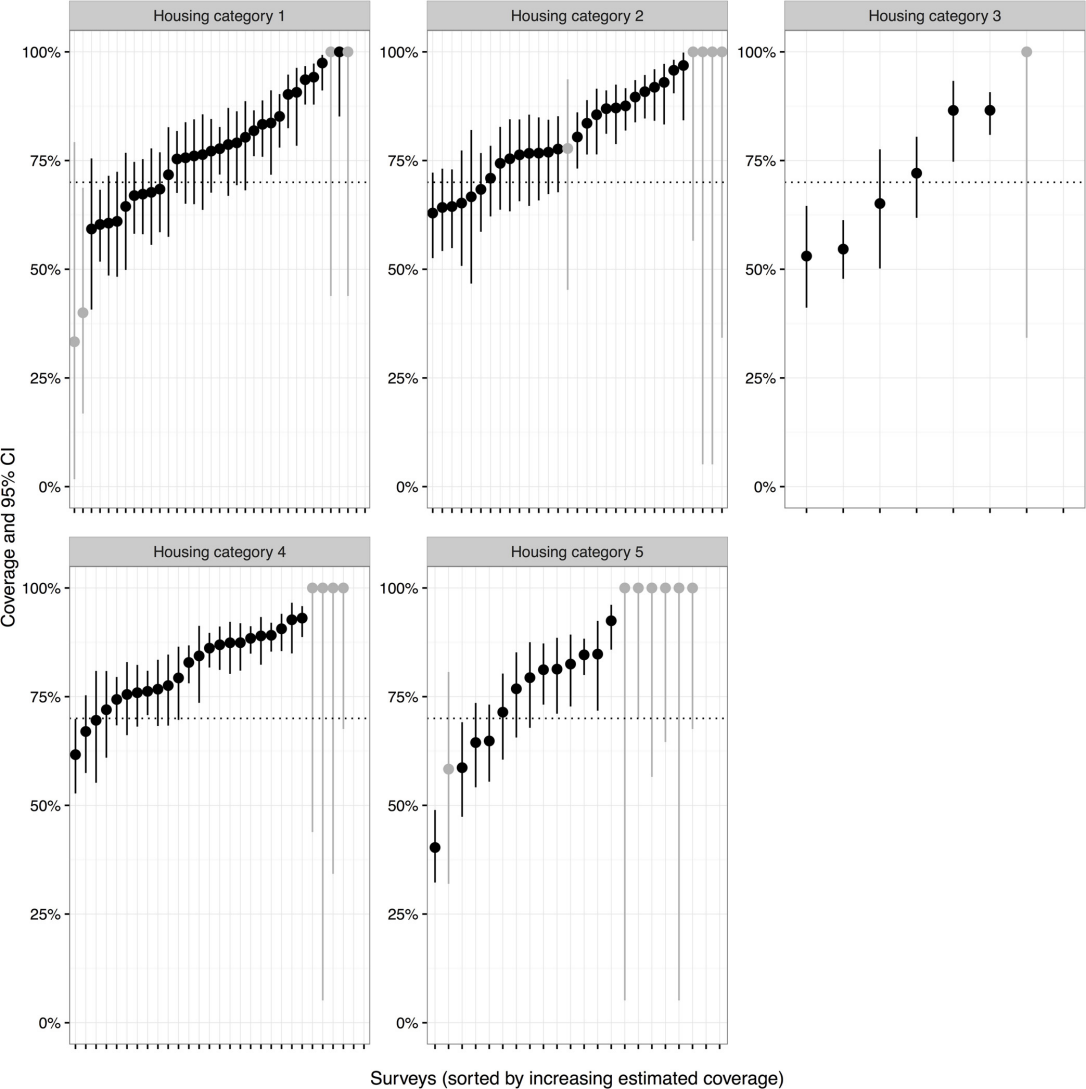
Graphs of estimated vaccination coverage per survey for all dogs (adults and puppies) by individual post vaccination survey and grouped according to Housing categories 1–5 with 95% confidence intervals. Light grey lines indicated surveys which recorded less than 20 dogs within that particular land type.

Surveys were conducted in 125 residential zones over a two week period immediately following the vaccination campaign. A total of 7,028 households were surveyed and the vaccination status of 10,919 dogs was ascertained (Table 3). Fifty-nine percent of households owned a dog. Of dog-owning households, an average of 1.93 adult dogs were owned. Mean number of dogs per household across the community (including non-dog owning households) was 1.55 dogs. The estimated vaccination coverage in the five main land types, based on 125 surveys, is shown in Table 3. For adult dogs, the estimated vaccination coverage was over 75% in all five land types with an overall coverage of over 85%. Similarly, the vaccination coverage for all dogs (adults and puppies) was over 75% in three land types and was only slightly below 75% in two (74.9% and 69.5%). The overall owned dog vaccination coverage for all dogs was 79.3% (Table 3). If it is assumed that no unowned dogs were vaccinated, and 97.1% of the population is owned, the overall coverage estimate is 77.4%, however this is conservative as some unowned dogs were vaccinated during door to door work. The slightly lower vaccination coverage of all dogs compared to the adult dog population alone was due to the lower vaccination coverage achieved in puppies (Table 3). Estimated coverage and confidence intervals for each of the individual post vaccination surveys in the five land types is shown for adult dogs (Fig 4), puppies (Fig 5) and all dogs (Fig 6). This shows that the vaccination coverage was consistently high across almost all surveys obtained. For example, only 10 of the 118 post-vaccination surveys in which adult dogs were observed had an estimated vaccination coverage of less than 70% (Fig 4). Furthermore, the estimated vaccination coverage was above 50% for 115 of the 118 surveys for all dogs (adults and puppies combined), although two of the surveys below 50% contained low sample sizes (Fig 5). S3 Dataset gives individual survey data and summarising tables.

**Table 3:** The table reports the coverage of the total number of vaccinated dogs in the housing category divided by the total number of animals in housing category. The 5th percentile is the 5th percentile of all individual survey coverages in that class & similar for 95^th^.

Housing Category	Group	Number of surveys	Total number of dogs	Median dogs recorded per survey	Overall coverage	% coverage 5 CI	% coverage 95 CI
1	dogs	35	2,617	66	77.6	51.6	96.8
1	adults	35	1,878	51	89.1	62.2	97.9
1	pups	35	739	18	48.4	0	92.7
2	dogs	31	2,499	85	80.7	64.3	100
2	adults	31	1,851	58	87	70.7	100
2	pups	31	648	22	62.5	15.3	95.8
3	dogs	8	640	59	69.5	53.5	91.9
3	adults	8	419	44.5	77.8	62.0	96.8
3	pups	8	221	16.5	53.8	29.3	76.2
4	dogs	29	3,799	116	82.9	67.8	100.0
4	adults	29	2,820	92	86.2	72.7	100.0
4	pups	29	979	21	73.3	30.3	97.4
5	dogs	22	1,364	66	74.9	57.4	100.0
5	adults	22	1,085	50.5	80.1	65.9	100.0
5	pups	22	279	9	54.5	6.8	97.8
**ALL**	**Dogs**	**125**	**10,919**	**78**	**79.3**	**78.6**	**80.1**
	**Adults**	**125**	**8,053**	**57**	**85.8**	**85.0**	**86.5**
	**pups**	**125**	**2,866**	**15**	**61.1**	**59.3**	**62.9**

### Demographics

Of all dogs vaccinated 57% were male. Of dogs presenting to static point clinics for vaccination, 14.5% were puppies, compared to 26.7% at door-to-door (p<0.001). (Table 4). Of all dogs that presented for vaccination, 10.4% of sterilized dogs were recorded as emaciated or underweight, compared to 22.8% of entire dogs (p<0.001).

**Table 4:** Age distribution of dogs stratified by static point and those vaccinated door-to-door vaccination efforts. Puppies were classified as approximately less than 3 months of age. This table does not include 365 static point vaccination records made on paper during the campaign.

Vaccination method	Adult	Puppy	Unknown	Total	% Puppies
Static point	19,705	3,354	18	23,442	14.5
Door-to-door	8,621	3,145	8	11,774	26.7
**Total**	**28,326**	**6,499**	**26**	**34,851**	

### Revised population estimate

The owned dog population of Blantyre was estimated at 44,261 (95% CI 43,893–44,630) using mark resight methods (Sheet 3 in S3 Dataset). The dog population consists of 97.1% owned dogs and so, following extrapolation for the unowned dog population, we estimate the total population of Blantyre City to be 45,526 (95% CI 45,147–45,906), giving a human:dog ratio of 18.1:1. Based on this estimated dog population of 45,526 dogs in Blantyre City, the initial pre-campaign dog sight surveys detected 42.4% of the dog population in the areas surveyed.

## Discussion

This study reports the systematic mass vaccination of dogs across a large African city, using smartphone technology to direct vaccination teams in the field and rapidly estimate vaccination coverage. Reports of successful large scale dog vaccination campaigns in Africa are currently scarce and previous efforts in Malawi have failed to achieve the coverage required to impact on disease transmission. This study adds to the growing evidence that the mass vaccination of dogs is possible and mobile technology can be used to address challenges in large scale project management.

A significant barrier to the elimination of rabies is the difficulty of developing and implementing vaccination programmes that enable large numbers of dogs to be vaccinated over a short space of time. For example, in a recent systematic review of canine rabies vaccination studies, the study which reported the largest number of vaccinated dogs in 29 articles, had a sample size of 4,271 dogs [7]. The methodology reported here demonstrated the feasibility of vaccinating a large number of dogs each day, with the vaccination of an average of 1,760 dogs per day during the 20 working days of vaccinations. A more gradual approach may have been applied [21], however, as was also reported during the vaccination of 9,000 dogs in two days in Lusaka, Zambia, an intensive period of vaccination across Blantyre city carried a number of advantages [30]. There was greater political engagement and support for a campaign which was conducted throughout the city over a shorter time period, which also enabled for the concentration of international expertise to establish methods and to train local staff. In the months following the intensive urban campaign, the established local team then continued to vaccinate across the large rural areas of Blantyre District. The intensive campaign in densely populated urban areas, coupled with a more protracted approach through the surrounding rural areas enabled effective distribution of resource and manpower throughout the year.

Another major barrier to the elimination of rabies is ensuring that a sufficiently high proportion of dogs have been vaccinated in order to break the cycle of rabies virus transmission amongst dogs. Annual vaccination of 70% of the dog population has been demonstrated to dramatically reduce the incidence of human and canine rabies cases after two cycles of vaccination [11,31], and free vaccination is often necessary to achieve adequate vaccination coverage in African settings [30,32]. This study reported a vaccination methodology which resulted in an estimated vaccination coverage in excess of 75% across the city. A recent study highlighted the importance of homogenous vaccination coverage, and that gaps in vaccination coverage may greatly hamper rabies elimination, even when mean coverage is high [9]. For logistical reasons, assessment of vaccination coverage was performed in the weeks following the Blantyre City campaign, at which point the temporary vaccination marks were already faded. Due to the high levels of dog ownership (97.1%), questionnaire surveys assessing coverage in the owned dog population provided a reliable estimate of overall vaccination coverage. These were conducted only in residential areas due to reliance on presentation of a vaccination certificate and the inaccessibility of many regions in the industrial/commercial and open space/agricultural land types. Additional post vaccination dog sight surveys in the days following vaccination would help to assess coverage in these less populated regions in the future. Sacrificing high coverage in some residential areas in favour of ensuring homogenous coverage in low density areas may hasten elimination of rabies from the city [9].

Estimating the size of the roaming dog population is essential to effective budgeting, vaccine procurement, purchasing of stock and recruitment of staff. The initial estimate generated from a reference human:dog ratio was sufficient for the planning of the campaign in this example, with subsequent refinement using mark-resight methods following conclusion of the work [29,33]. The estimated human:dog ratio for Blantyre City generated from this campaign (18.1:1) is comparable to reports from other urban study sites in sub-Saharan Africa [13,24,34,35], and supports the use of human:dog ratios as a rapid and inexpensive method of initial population estimation where human census data is available. It is important to consider cultural and geographic factors which may vary between locations and can have a significant impact on local human:dog ratios [7,34].

In this study, dog sight surveys were used to assess the distribution of dogs across the city and therefore improve the strategic approach to vaccination, as well as to refine the dog population estimate. The population estimate calculation did not include the ‘detectability’ of the survey; the proportion of the total population that are detected through the methods of the survey [36]. Detectability can be assessed through comparison with a more intensive survey method [37] and in this instance was estimated to be 42.4% using the vaccination campaign as a mark-resight survey. Although published reports describing roaming dog detectability are lacking, the estimate in this report is similar to studies elsewhere [36,38]. Further research is needed to assess variation in roaming dog detection across different local settings and between survey methods. This example emphasises that population estimates generated using dog sightings alone, without accounting for detectability, should not be used as the basis for estimating the vaccination coverage achieved by a campaign.

The 2014 government vaccination campaign using only static point vaccination achieved an estimated vaccination coverage of 37% across Blantyre District (urban and rural) [23]. However, during the present study, observations made during the door-to-door vaccination programme found that only 42.4% of dogs were accessed through static point vaccination. This observation provides compelling evidence that static points alone are insufficient to ensure high vaccination coverage in urban Malawi. Static point vaccination is a more efficient method of vaccination than door-to-door, however its success relies on a high proportion of dogs being presented by the public, which in turn depends on social, cultural, geographic and economic factors [14]. The need for additional door to door vaccination has also been demonstrated in in semi-rural Zambia, suburban Kenya and pastoral communities in Tanzania [12,39] and in some settings may be the only way to reach the vaccination coverage required to eliminate rabies [14]. Therefore, developing door-to-door vaccination methods which can be implemented with greater logistical ease and efficiency may help to improve the cost-effectiveness and scalability of such campaigns. Economic analysis of this campaign is beyond the scope of this paper, however analysis is currently underway to compare the cost-effectiveness of this method with other approaches.

The use of mobile technology in this study not only enabled the systematic direction of vaccination teams in the field, but also the rapid assimilation of data. The ability for teams to navigate within a defined boundary via a smartphone application in areas with no distinct landmarks or road names meant that thorough, systematic door-to-door vaccination was possible without local geographic knowledge. The time saved in converting paper records to digital spreadsheets resulted in savings in staff time, increased speed of data interpretation and real-time field crew management. The benefits of mobile technology in field data collection have been recognised for some time [18], however this is the first report of mobile technology being applied specifically to facilitate mass dog vaccination in Africa. In addition to improving the management and direction of the field teams; collection, analysis and presentation of GPS data aided in the rapid visual presentation of work to stakeholders and aided the explanation of the scale of the problem and necessary intervention to interested parties.

Presentation of puppies to static point was lower than the proportion vaccinated at door-to-door vaccination, highlighting the need for public education that it is safe and necessary to vaccinate dogs of all ages [40]. It may also be more logistically challenging for dog owners to carry litters of puppies on foot to a static point vaccination station, which will also be the case in other parts of Africa. The mortality rate in puppies in Blantyre is not known, however the lower vaccination coverage achieved in the puppy population may result in a more rapid dilution of vaccination coverage as unvaccinated individuals become adults. Exploration of alternative methods of accessing puppies for vaccination may help to increase coverage achieved in this important and often under-vaccinated sub population.

A further fundamental barrier to the success of a rabies control program is the coordination of stakeholders. In many developing countries, rabies control initiatives on the scale required to impact on disease incidence are beyond the financial or resource capabilities of the government; however international contributions can provide the possibility of improving the situation. A Rabies Focus Group was established in Blantyre following the publication of human data from the Queen Elizabeth Central Hospital and has brought together stakeholders from the national and local government, human and veterinary sectors. The group stimulated discussion on the wider implications of rabies and brought international collaboration focus on mass dog vaccination. As well as continuing to vaccinate in Blantyre District, this team will be used to build the work force through training others in these methods. Additional sustainable funding sources need to be explored in order to expand this work in building towards a national rabies elimination strategy over the coming years.

This is the first study to report on the demographics of a dog population in Malawi. Dog ownership in 59% of households is similar to that reported in a study in Kenya [41], but higher than urban ownership in a study in Zambia [39]. The male:female ratio of 56:44 is comparable to many other studies of dog populations in developing countries [7]. Neutered dogs were significantly less likely to be emaciated or underweight than entire dogs, which is consistent with findings in other developing countries [42]. Efforts to promote responsible dog ownership and improve the health of the population, educating the community as to the benefits of neutering and targeting its application at those dogs which are producing unwanted puppies will help to achieve a healthier, more stable dog population.

The use of bright yellow uniforms during the vaccination programme, a large scale concurrent school education programme and involvement of international volunteers increased the visibility of the vaccination work within the community and not only increased awareness of rabies vaccination, but also helped to instil a sense of pride and purpose within the project staff. A staff engagement session was incorporated into initial training days in order to listen to the local team’s perception of rabies and engage their views on how the problem can be best addressed. The presence of international volunteers brought a dynamic atmosphere to the work and, through their fundraising efforts, contributed to the funding of the programme. In addition to this, the volunteers served to train local staff in standard operating procedures, data collection and humane animal handling methods.

Further study is required to investigate the longer term impact of this initiative on human and canine rabies incidence in Blantyre, as well as the cost effectiveness of such a campaign over other methods of control. Innovation of new project structures and utilisation of emerging technologies enable the development of more efficient methods of mass dog vaccination; working towards the elimination of canine transmitted rabies. Rabies is endemic across a wide spectrum of cultures and although each location needs an approach which is tailored to the local challenges, this method may provide a number of advantages in mass dog vaccination initiatives elsewhere.

### Conclusion

The authors believe that the elimination of canine transmitted rabies is absolutely achievable, with this study demonstrating that mass vaccination of dogs, to a high coverage in the urban African setting is possible. Communities are able to present a sufficient proportion of animals for vaccination in order to control the disease, however the co-ordination and mobilization of resources required to conduct a campaign on a sufficiently large scale can be challenging. As demonstrated for the first time in this study, the use of mobile technology in data collection and team management is particularly powerful in the mass dog vaccination setting and although it may not be suitable for use in all settings, provides huge potential for coordinating and monitoring multiple teams from a central location.

## Supporting Information

S1 Dataset Excel file containing spreadsheet of city zones, area and number of sighted dogs on initial dog sight surveys by confinement. (XLSX)

S2 Dataset Excel file containing four sheets; Sheet 1 contains individual dog vaccination records; Sheet 2 contains data clustered by individual static point clinics and date; Sheet 3 contains vaccinations door-to-door data clustered by land type and date; Sheet 4 contains tables summarising figures. (XLSX)

S3 Dataset Excel file containing three sheets; Sheet 1 contains individual post vaccination survey dog data; Sheet 2 contains summary of calculations from post vaccination surveys; Sheet 3 contains a summary of calculations for mark-resight figures. (XLSX)
